# No evidence for zoonotic transmission of H3N8 canine influenza virus among US adults occupationally exposed to dogs

**DOI:** 10.1111/irv.12208

**Published:** 2013-11-15

**Authors:** Whitney S Krueger, Gary L Heil, Kyoung-Jin Yoon, Gregory C Gray

**Affiliations:** a Emerging Pathogens Institute and College of Public Health and Health Professions, University of FloridaGainesville, FL, USA; b College of Veterinary Medicine, Iowa State UniversityAmes, IA, USA

**Keywords:** Communicable diseases, emerging, Dog diseases, influenza A virus, occupational exposure, seroepidemiologic studies, zoonoses

## Abstract

**Objectives:**

The zoonotic potential of H3N8 canine influenza virus (CIV) has not been previously examined; yet considering the popularity of dogs as a companion animal and the zoonotic capabilities of other influenza viruses, the public health implications are great. This study aimed to determine the seroprevalence of antibodies against CIV among a US cohort.

**Design:**

A cross-sectional seroepidemiological study was conducted between 2007 and 2010.

**Setting:**

Recruitments primarily occurred in Iowa and Florida. Participants were enrolled at dog shows, or at their home or place of employment.

**Sample:**

Three hundred and four adults occupationally exposed to dogs and 101 non-canine-exposed participants completed a questionnaire and provided a blood sample.

**Main outcome measures:**

Microneutralization and neuraminidase inhibition assays were performed to detect human sera antibodies against A/Canine/Iowa/13628/2005(H3N8). An enzyme-linked lectin assay (ELLA) was adapted to detect antibodies against a recombinant N8 neuraminidase protein from A/Equine/Pennsylvania/1/2007(H3N8).

**Results:**

For all assays, no significant difference in detectable antibodies was observed when comparing the canine-exposed subjects to the non-canine-exposed subjects.

**Conclusion:**

While these results do not provide evidence for cross-species CIV transmission, influenza is predictably unpredictable. People frequently exposed to ill dogs should continually be monitored for novel zoonotic CIV infections.

## Introduction

In the last decade, H3N8 canine influenza A virus (CIV) has rapidly spread through the US dog population. First isolated in 2004 from racing greyhounds experiencing respiratory disease at tracks in Florida, CIV emerged from a rare antigenic drift in the H3N8 equine influenza virus that resulted in direct horse-to-dog transmission without viral reassortment.^1^ Previous to this, dog-to-dog transmission of influenza viruses was not thought to occur.^2^ This new virus had a novel genetic makeup efficient in spreading dog-to-dog.^3^

CIV outbreaks affected thousands of racing greyhounds and were subsequently identified in animal shelters 8 months after its discovery.^1^,^2^,^4^ With a high attack rate and respiratory symptoms similar to “kennel cough”, in May 2009, the United States Department of Agriculture approved a CIV killed-virus vaccine for dogs that proved to decrease the spread, signs, and severity of infection.^5^ CIV has been confirmed in the majority of US states and is considered an enzootic pathogen in Florida, New York, Philadelphia, and Colorado, with additional case clusters in New Jersey and Wyoming.^1^,^3^,^6^ As with other influenza viruses, its evolution is unpredictable, and viral changes must be continually monitored as it moves through the dog population.^4^

Worldwide, dogs are a popular companion animal, especially in the United States; however, with 78·2 million dogs owned in the United States and 40% of US households owning at least one dog,^7^ this popularity has public health implications. The approximately 5000 US animal shelters are increasingly more crowded, with an average of 6 dogs entering a given shelter every day.^8^ Overcrowded shelters and kennels create an ideal environment for amplified infectious disease transmission among dogs. Furthermore, infectious diseases flourishing among overcrowded dogs present a precarious opportunity for zoonotic pathogens to emerge and threaten people who work in close contact with dogs.

Human CIV infections have never been reported or studied; however, humans have been shown to be infected with influenza viruses that circulate in birds, pigs, and horses.^9^,^10^ Moreover, human influenza viruses have been shown to infect dogs.^11^–^14^ As the very similar H3N8 equine influenza virus strains have been shown capable of infecting humans,^15^–^17^ it seems prudent to ask if CIV is also zoonotic. Canine cases have been linked to dog racetracks, kennels, and shelters^18^ where substantial numbers of dogs are housed together; therefore, employees working at these facilities are likely exposed to CIV. In this cross-sectional seroepidemiological study, we sought to identify subclinical and unapparent CIV infections in a highly dog-exposed US adult population.

## Materials and methods

### Participant recruitment and enrollment

The study was approved by the University of Iowa and the University of Florida's institutional review boards. The target population included dog breeders, kennel employees, veterinary personnel, animal shelter workers, greyhound racetrack employees, and dog show handlers whose work or hobby involved exposure to multiple dogs. A non-canine-exposed, non-matched control group was drawn from a convenience sample of individuals affiliated with the University of Iowa or University of Florida, who had neither been exposed to multiple dogs as part of their work or hobby, nor had pet dogs in their household, in the last 5 years. All participants had to be at least 18 years of age and self-report no current immunocompromising conditions.

Breeders, kennels, and veterinary clinics were identified through state databases of licensed breeders and practicing veterinarians. Local shelters, clinics, and greyhound racetracks were also identified through internet searches. Canine-exposed subjects were recruited via mailed letters, telephone calls, and face-to-face encounters; enrollments typically occurred at their home or place of employment, but also at local dog shows and trade shows. Non-canine-exposed subjects were recruited via face-to-face encounters in university common areas frequented by faculty, staff, and students. After informed consent was obtained, all participants completed a self-administered questionnaire and permitted collection of a blood specimen. The enrollment questionnaire documented participants' demographics, frequency of exposure to domestic animals, and whether subjects had ever been exposed to a dog manifesting signs of kennel cough or known to be infected with CIV. Canine-exposed participants completed an additional questionnaire section to ascertain details of their occupations/hobbies that involved exposure to dogs and personal hygiene practices when working with dogs.

### Laboratory methods

Whole blood specimens were transported on ice to the laboratory within a few hours of collection. Blood tubes were spun at 3000×g for 15 minutes at room temperature to separate serum. All collected serum was aliquoted and frozen at −80°C. The microneutralization (MN) and neuraminidase inhibition (NI) assays were performed to detect serum antibodies against the A/Canine/Iowa/13628/2005(H3N8) influenza virus.^19^ An enzyme-linked lectin assay (ELLA) designed to detect subtype-specific antibodies against the catalytic site of the NA protein was also adapted against a recombinant N8 neuraminidase protein from A/Equine/Pennsylvania/1/2007(H3N8) influenza virus as the antigen. In addition, the hemagglutination inhibition (HI) assay was performed to detect potentially cross-reacting antibodies against the A/Brisbane/10/2007(H3N2) human influenza virus. Influenza viruses were grown in 10-day-old embryonated chicken eggs (Charles River Laboratories, Wilmington, MA, USA). After 4 egg passages, the H3N8 CIV was then propagated in Madin–Darby canine kidney (MDCK) cells to achieve a higher virus titer.

#### Hemagglutination inhibition (HI) assay

Following previous reports,^20^,^21^ a quantitative HI assay was used to examine subjects' sera for antibodies with hemagglutinin (H3) subtype specificity using the Centers for Disease Control and Prevention (CDC) HI assay protocol against an H3N2 human influenza A virus (A/Brisbane/10/2007 [CDC]) with a 0·65% suspension of guinea pig erythrocytes. Each serum sample included a negative control well with no virus, and assay controls of positive H3 antisera and uninfected sheep serum from a recent World Health Organization Influenza Reagent Kit were also included.

#### Microneutralization (MN) assay

Following previous reports,^22^–^28^ an adapted MN assay^29^ was performed using the A/Canine/Iowa/13628/2005(H3N8) influenza virus. Sera were heat inactivated at 56°C for 30 minutes and screened at a 1:10 dilution in duplicate. Sera with at least one duplicate at an antibody titer of 1:10 were retested in duplicate with a twofold serial dilution up to 1:1280. Canine sera from dogs with known CIV infections (and pre-tested to determine HI and MN assay antibody titers) were used as positive controls.

#### Neuraminidase inhibition (NI) assay

Based on previous reports,^30^–^32^ a qualitative NI assay was developed to examine subjects' sera for antibodies with neuraminidase (N8) subtype specificity using A/Canine/Iowa/13628/2005(H3N8) as the whole virus antigen. A virus titration was first performed to determine the optimum antigen dilution, which was the highest dilution with a “medium” pink color or the dilution prior to a reduction in pink color. Sera were heat inactivated at 56°C for 30 minute then diluted 1:2·25 in phosphate-buffered saline (PBS), per previous reports.^31^ Using white opaque polystyrene 96-well microtiter plates (Nunc, Rochester, NY, USA), 25 μl of test sera was added in duplicate, followed by 25 μl of standardized virus. Virus control wells with PBS only as well as positive control wells employing canine sera were used as assay controls (note, due to their high reactivity, canine control sera were diluted at 1:64). The NI assay was then followed as previously described.^31^ A dark pink color was deemed negative, and a reduced pink or light pink color was considered positive. Positive results were classified as strong positive or weak positive, based on the level of color reduction.

#### Enzyme-linked lectin assay (ELLA)

An ELLA previously described by Hassantoufighi, *et al*.^33^ was adapted to use a baculovirus expressed recombinant neuraminidase subtype 8 (rN8) (BEI Resources catalog #NR-13523) from the A/Equine/Pennsylvania/1/2007(H3N8) influenza virus, which is 99% homologous to CIV strains. The soluble rN8 used in this assay was demonstrated by the supplier to be functionally active based on its ability to cleave the fluorogenic substrate 2′-(4-methylumbelliferyl)-α-D-N-acetylneuraminic acid (4-MUNANA), indicating it would be appropriate for use in a functional neuraminidase inhibition assay.

In addition to using rN8 protein in place of whole viral antigen, the ELLA procedure described by Hassantoufighi^33^ was also modified by implementing a screening test at 1:10 in duplicate. Sera testing positive at the 1:10 dilution were subjected to a titration assay in which serial twofold dilutions of the specimen were tested in duplicate. A goat polyclonal antiserum to the N8 of A/Equine/Miami/1/63 (BEI Resources NR-3145) was included at 1:20 on screen plates and as a single serial twofold dilution (1:20–1:2560) on titration plates.

A well was determined to be positive for N8-specific inhibiting antibodies when the absorbance levels were <50% of the average of the standardized antigen control wells (contained no serum) for the corresponding plate after background levels (average of plate control wells which contained only sample diluent) were subtracted from all wells on the plate.

The standardized concentration of rN8 used for this assay was determined by performing the assay described above with multiple replicates of serially diluted antigen in the absence of any serum specimen. The dilution of stock antigen was selected as the dilution which produced absorbance levels at approximately 80% of saturation under the assays conditions.

### Statistical methods

The chief independent variable, canine exposure, was examined in a number of ways: dichotomized (exposed/unexposed), categorical, and continuous (dog-years of exposure). Occupations/hobbies were subgrouped as breeder, kennel owner, kennel employee, veterinarian, veterinary staff, shelter worker, groomer, trainer, racetrack employee, and “other”. For each occupational subgroup, subjects reported the number of years they had participated in that occupation/hobby as well as the average number of dogs they are/were exposed to in that given occupation/hobby. From these numbers, the continuous variable of dog-years of exposure was calculated for each occupational subgroup by multiplying the two variables. Because participants were allowed to cite more than one occupation/hobby, overall dog exposure was calculated by adding the dog-years of exposure for each occupational subgroup.

The study outcome was serological evidence of previous infection with CIV by the various assays (MN, NI, and ELLA) run on participants' sera. Because of a low prevalence of elevated antibodies against CIV and our inability to determine in this cross-sectional analysis when such an infection might have occurred, we chose a low MN and ELLA titer threshold (≥1:10) as evidence of previous infection with CIV. Because cross-reactions from previous infection with human viruses might confound CIV serology, we sought to control for this confounding by adding human influenza virus serological reactivity covariates to the multivariate models when the bivariate analyses suggested they were important outcome predictors. Per CDC guidelines, previous infection with human influenza H3N2 virus was defined as having an HI titer ≥1:40.^34^

Logistic regression was used to compare seroreactivity between canine exposure groups and ascertain odds ratios and associated 95% confidence intervals. To maximize the power to detect differences between the exposure groups, the proportional odds model^35^ was used in assessing associations with ordinal MN, NI, and ELLA outcomes when the proportional odds assumption was met. Comparisons between exposure groups were also made after adjusting for potential confounders such as age, prior influenza vaccination, and elevated HI titer against human H3N2 virus. Analysis was performed using sas v9.2 (SAS Institute, Cary, NC, USA).

## Results

Between 2007 and 2010, 304 canine-exposed and 101 non-canine-exposed subjects were enrolled (Table 1). Demographically, the gender distribution was identical between exposure groups; however, the controls tended to be younger than the exposed group (mean of 33yo and 43yo, respectively). Overall, the participants were more likely to be female (68%), and 75% resided in Iowa or Florida where the majority of enrollments took place. Table 2 illustrates the participants' occupations/hobbies involving close contact (approximately 3 ft) with dogs (respondents were allowed to report more than one). On average, a single given occupation/hobby involved a median of 150 dog-years of exposure.

**Table 1:** Unadjusted odds ratios for demographics and serological results for canine-exposed participants compared with non-canine-exposed participants using binary logistic regression

Covariate	*n* (*N* = 405)	Exposed (*n* = 304)	Control (*n* = 101)	Unadjusted OR (95% CI)
Age (years)*				
49–78	136	120 (39·6)	16 (16·0)	6·4 (3·4–12·0)
30–48	137	113 (37·3)	24 (24·0)	4·0 (2·3–7·1)
18–29	130	70 (23·1)	60 (60·0)	Ref
Gender				
Male	129	97 (31·7)	32 (31·7)	1·0 (0·6–1·6)
Female	276	207 (68·1)	69 (68·3)	Ref
Level of education*				
Graduate college	93	49 (16·2)	44 (43·6)	0·1 (0·1–0·3)
2–4 year college /Professional school	215	168 (55·5)	47 (46·5)	0·4 (0·2–0·9)
High school or less	96	86 (28·4)	10 (9·9)	Ref
Ever used tobacco products*				
Yes	119	99 (34·7)	20 (19·8)	2·2 (1·2–3·7)
No	267	186 (65·3)	81 (80·2)	Ref
Exposed to horses*				
Yes	286	244 (82·2)	42 (45·2)	5·6 (3·4–9·3)
No	104	53 (17·7)	51 (54·8)	Ref
Ever received human influenza vaccine*				
Yes	229	160 (55·9)	69 (69·7)	0·6 (0·3–0·9)
No	156	126 (44·1)	30 (30·3)	Ref
Human H3N2 influenza titer*				
≥1:40	106	66 (21·9)	40 (40·0)	0·4 (0·3–0·7)
<1:40	296	236 (78·2)	60 (60·0)	Ref
H3N8 Microneutralization assay titer				
≥1:10	76	63 (20·7)	13 (12·9)	1·8 (0·9–3·4)
<1:10	329	241 (79·3)	88 (87·1)	Ref
H3N8 Neuraminidase inhibition assay*				
Positive	74	58 (19·1)	16 (15·8)	1·3 (0·7–2·3)
Negative	330	245 (80·9)	85 (84·2)	Ref
H3N8 Enzyme-linked lectin assay*				
Positive	18	14 (4·6)	4 (4·1)	1·1 (0·4–3·6)
Negative	382	288 (95·4)	94 (95·9)	Ref

* Covariate has some missing data.

**Table 2:** Occupational and hobby exposures as cited by subjects

Occupation*	*n*	Median dog-years of exposure (IQR)**^,^***
Veterinary staff	91	78 (24–200)
Breeder	88	60 (25–233)
Kennel worker	97	80 (30–300)
Veterinarian	63	140 (80–264)
Shelter staff	47	54 (16–160)
Trainer	39	50 (12–160)
Groomer	23	50 (14–210)
Racetrack staff	16	540 (200–1560)
Dog show handler	12	60 (26–186)
Owner/Hobbyist	7	50 (18–90)
Researcher	2	19 (5–32)
Pet store staff	1	180 (180–180)

* Subjects allowed to cite multiple occupations.

** Calculated as the reported number of years multiplied by the average number of dogs per day.

*** Interquartile range.

### Seroprevalence findings

**Figure 1:**
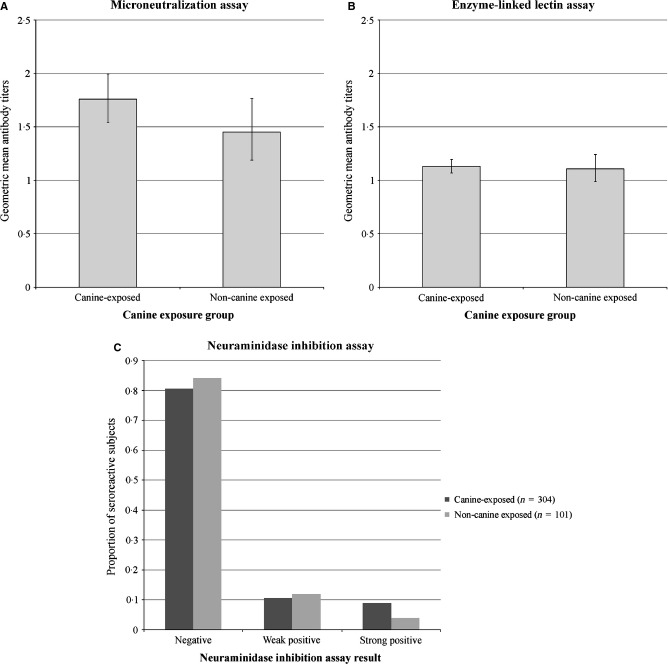
Comparison of seroreactivity by exposure group, for various serological assays: (A) Geometric mean titers for the microneutralization assay (B) Geometric mean titers for the enzyme-linked lectin assay (C) Seroreactivity proportions for the neuraminidase inhibition assay. MN and ELLA antibody titers <1:10 were assigned the value of 1 for calculation purposes.

For all three serological assays (MN, NI, and ELLA), there was no significant difference in elevated antibody titers between the canine-exposed and non-canine-exposed subjects (Figure 1). A total of 76 subjects had a MN assay titer ≥1:10, of which 63 (20·7%) were canine-exposed subjects compared with 13 (12·9%) seropositive non-exposed subjects (OR = 1·7; 95% CI, 0·9–3·3). In addition, participants who tested seropositive (titer ≥1:40) for human H3N2 influenza (OR = 1·8; 95% CI, 1·1–3·1) and participants who reported ever receiving a human influenza vaccine (OR = 1·9; 95% CI, 1·1–3·3) were significantly more likely to have MN titers ≥1:10 against CIV. For the NI assay, 75 subjects were classified as positive: 31 strong positives (27 [8·9%] canine-exposed and 4 [4·0%] non-exposed) and 44 weak positives (32 [10·5%] canine-exposed and 12 [11·9%] non-exposed) (OR = 1·3; 95% CI, 0·7–2·4). Reporting ever receiving a human influenza vaccine was significantly associated with a positive NI result (OR = 2·2; 95% CI, 1·2–4·0). Eighteen subjects had an ELLA titer ≥1:10, of which 14 (4·6%) were canine-exposed and 4 (4·0%) were non-canine-exposed (OR = 1·1; 95% CI, 0·4–3·6). Prior vaccine receipt and elevated human H3N2 influenza titer were not associated with a positive ELLA result.

Interassay agreement did not well correlate. The respective Cohen's kappas were as follows: NI and ELLA (0·3); MN and NI (0·05); MN and ELLA (−0·01).

## Discussion

Our serological study did not support the premise that CIV is a zoonotic pathogen; nonetheless, this study was limited in a number of ways. The MN assay is the most accepted method of detecting human antibodies against novel animal-origin influenza viruses,^36^ and increased odds of elevated antibodies for canine-exposed subjects by the MN assay narrowly missed the 0·05 level of significance (*P* = 0·09). A larger sample size may have provided the necessary power to detect sporadic cases of CIV infections in humans. While we could not implicate overall dog exposure as associated with increased antibodies against CIV, we also could have missed an important occupational exposure within a subgroup (e.g., shelter workers), where our data were too sparse to detect a significant association with the study outcome.

In addition, the lack of prevalence data for CIV infections among dogs in the study areas weakened the strength of this study. Without prevalence data in the canine population, it is difficult to distinguish if negative results indicated CIV was circulating among dogs but not causing zoonotic infections, or if people were not being exposed to the virus in the first place. A seroprevalence study of CIV in US pet and shelter dogs conducted between 2005 and 2009 which overlapped with much of this study's enrollment period found that in Florida, 187 (42%) of 444 dogs tested from 119 locations were seropositive for H3N8 CIV antibodies.^37^ While Iowa was not specifically examined, the authors reported an overall seroprevalence in the Midwest to be 11% (6/56) among dogs tested from 19 locations. CIV data specific to dogs in Iowa were presented in a 2009 seroprevalence study of blood samples submitted to Iowa State University from a convenience sample of 731 dogs (84% were clinically ill at sampling, but only 1% presented with a respiratory disease).^38^ Four dogs (<1%) had evidence of antibodies against H3N8 CIV. The limited resources afforded to this present study did not permit simultaneous sampling of the dogs cared for by the study participants.

Another key limiting factor in our study was the likely presence of cross-reacting antibodies. Infection with one influenza virus can render a person immune to attack by a closely related virus with similar surface glycoproteins. In serological diagnoses, this is an obstacle difficult to overcome and achieve virus specificity. Because completely controlling for cross-reacting antibodies is often unachievable, this study employed a non-exposed comparison group, considered prior receipt of influenza vaccines as well as infection with human H3N2 influenza virus in statistical analyses, and performed three serological assays to corroborate results.

Because no human influenza virus containing the N8 protein is known to exist, the NI assay was employed as a means to validate the MN assay results, by using the same A/Canine/Iowa/13628/2005(H3N8) influenza isolate. The only known NI assay cross-neutralization has been documented between NA1 and NA4 specific antibodies^31^; however, reaction with other NA-specific antibodies with the NA8 may be occurring. This may be especially true for human influenza viruses that circulated decades ago, such as the human H2N2 influenza virus. In addition, it has been documented in NI assays employing whole virus as the antigen that high antibody titers against the HA antigen can cause inhibition of neuraminidase activity via non-NA-specific steric hindrance mechanisms.^39^

To further examine possible evidence of CIV infection while controlling for potential cross-reactivity with human HA antibodies, we adapted an ELLA that detected neuraminidase inhibition activity against a recombinant N8 protein. This likely improved assay specificity, as ELLA seropositivity was not associated with human influenza infections or vaccines. Detection of heterosubtypic NA neutralizing antibodies^40^,^41^ by this assay is one possible explanation as to why the anti-N8 response observed in the functional-based NA assays does not correspond to the MN data.

Another limitation to this study was that identifying a truly non-canine-exposed control group would be nearly impossible with the popularity of dog ownership in the United States. An exposure cut-off of 5 years was selected to limit the potential of recent canine exposures to confound study results. Because of the seropositivity among the control group, it cannot accurately be determined whether antibodies against other influenza viruses were confounding serological results or whether, in fact, these subjects mounted a serological response when incidentally exposed to dogs in the present or more than 5 years ago. Comparisons between the exposure groups may have been more accurate if the canine-exposure cut-off was increased to 10–20 years, as well as if lifetime dog exposure data were collected from all study participants.

Human infections with CIV may be occurring at a low level of incidence, for which this sample size was not large enough to detect a significant difference between the exposure groups (power <0·5) by means of serology. While seroepidemiological studies have their limitations, they are often used as a first step in examining evidence for novel zoonotic virus transmission. Despite the negative results of this study, the propensity of influenza viruses to mutate and reassort suggests that CIV could gain the ability to infect humans in the future. Continued research is warranted to monitor the zoonotic potential of CIV.
